# An Innovative Virtual Reality System for Measuring Refractive Error

**DOI:** 10.3390/diagnostics14151633

**Published:** 2024-07-29

**Authors:** Chin-Te Huang, Chien-Nien Lin, Shyan-Tarng Chen, Hui-Ying Kuo, Han-Yin Sun

**Affiliations:** 1 Department of Ophthalmology, Chung Shan Medical University Hospital, Taichung 40201, Taiwan; 2 Department of Ophthalmology, School of Medicine, College of Medicine, Chung Shan Medical University, Taichung 40201, Taiwan; 3 Department of Optometry, College of Medical Science and Technology, Chung Shan Medical University, Taichung 40201, Taiwan

**Keywords:** virtual reality, refractive error, light field technology, eye examination, vision assessment, optical care

## Abstract

In this study, we aimed to validate a novel light field virtual reality (LFVR) system for estimating refractive errors in the human eye. Fifty participants with an average age of 22.12 ± 2.2 years (range 20–30 years) were enrolled. The present study compared spherical equivalent (SE) and focal line measurements (F1 and F2) obtained by the LFVR system with those obtained by established methods, including closed-field and open-field autorefractors, retinoscopy, and subjective refraction. The results showed substantial agreement between the LFVR system and the traditional methods, with intraclass correlation coefficients (ICC) for SE ranging from 82.7% to 86.7% (*p* < 0.01), and for F1 and F2 from 80.7% to 86.4% (*p* < 0.01). Intra-repeatability for F1 and F2 demonstrated strong agreement, with ICC values of 88.8% and 97.5%, respectively. These findings suggest that the LFVR system holds potential as a primary tool for refractive error measurement in optical care, offering high agreement and repeatability compared to conventional methods.

## 1. Introduction

Globally, uncorrected refractive error (URE) is the leading cause of vision impairment, followed by cataracts and age-related macular degeneration [1,2]. Approximately 116 million instances of moderate to severe vision impairments can be attributed to URE, which can adversely affect the education and employment opportunities of affected individuals [3]. Alarmingly, more than half of the global population is projected to be impacted by myopia [4], with prevalence rates ranging from 84% to 97% in Asian countries such as Korea, Taiwan, and Singapore [5,6]. This myopia epidemic, affecting over 80% of the population in these regions, exerts a substantial socio-economic impact, particularly on younger demographic cohorts in urban areas. URE has been estimated to cost the global economy around $269 billion [7]. High myopia increases the risk of pathological degeneration, such as glaucoma, retinal detachment, chorioretinal atrophy, and lacquer cracks, potentially leading to irreversible vision loss [8].

Early detection of myopia in children and adolescents is crucial in averting the pathological complications associated with high myopia. Significant URE during childhood can also contribute to conditions such as amblyopia and strabismus, thereby highlighting the importance of regular visual screening to prevent vision impairment. However, conducting mass visual screening poses a considerable challenge, especially in geographically large and diverse nations. In developing regions, such as Sub-Saharan Africa and India, the availability of eye care professionals is alarmingly low, with only 2.5 eye doctors [9] and 1.6 optometrists per million individuals [10], respectively. The development and availability of rapid and precise diagnostic tools aid optometrists and eye care professionals in gathering critical data. Measurement of refractive error, widely utilized in clinical optometry, provides essential information for eye care practice [11,12]. High-quality autorefractors, offering high repeatability and agreement, furnish clinicians with insight into patients’ refractive error, hence enabling enhanced patient care. Their rapid and accurate measurement capabilities have made autorefractors an indispensable tool for optometrists.

Various techniques have been developed to quickly screen and accurately measure the ocular sphere-cylindrical refractive value, an initial step in assessing subjective refraction (SR) in clinical applications. These techniques encompass the gold standard, retinoscopy (RET), an assortment of autorefractors, and the wavefront aberrometer. Numerous autorefractor types, including open-field and closed-field autorefractors (OFA and CFA), are widely recognized as reliable and valid objective refractive tools within the field of optometry [13,14]. However, several hurdles persist in the implementation of autorefractors. For instance, closed-field autorefractors often report an additional myopic refraction of about 0.3 to 0.5 D compared to open-field autorefractors and retinoscopy [15,16]. This discrepancy is attributed to the accommodative instability caused by instrument-induced myopia. Moreover, traditional autorefractors demonstrate lower efficacy in detecting ocular pathologies such as cataracts and keratoconus compared to retinoscopy and wavefront analyzers [17,18,19]. Wavefront autorefractors, while offering both low-order (refractive power) and high-order aberration measures, are associated with higher costs [20,21].

Vision screening on a mass scale necessitates precision, user-friendliness, and economic efficiency in the tools utilized. Furthermore, this process often involves the enlistment of non-specialist personnel, including school nurses, teachers, and community healthcare workers, to conduct these screenings [22]. Consequently, the development of cost-effective, user-friendly, and accurate tools for the estimation of refractive error is integral for facilitating a capable workforce for these mass vision screening initiatives [23]. With the growing prominence of computer-based technologies, such as augmented reality (AR) and virtual reality (VR), a new frontier for refractive error measurement has been introduced through VR applications. Yet, VR and AR continue to grapple with the persistent challenge of vergence-accommodation conflict (VAC). To overcome this issue, the utilization of a near-eye display (NED) system equipped with a light field is advantageous, as it mitigates VAC in VR applications and yields superior depth accuracy at proximate distances [24].

The aim of this study was to validate the accuracy of light field virtual reality (LFVR) technology in comparison to established clinical methods for measuring refractive error. This study integrated the use of RET, CFA (Topcon KR800S; Topcon Medical Systems, Inc., Oakland, NJ, USA), OFA (Shin-Nippon Nvision-K 5001, Takamatsu-shi, Kagawa-ken, Japan), SR, and LFVR in the experimental design. The primary objective was to measure refractive error using the VR system, an innovative approach designed for accuracy within constrained spaces and equipment limitations. The ultimate aim was to ensure the final measurement equated to the clinically accepted gold standard of SR. The findings indicate that LFVR technology is a viable and accurate alternative to traditional refraction tests. Moreover, the data reveal the potential for LFVR technology to augment the user experience within VR applications.

## 2. Materials and Methods

### 2.1. Participants

This study was approved by the Ethics Committee of Chung Shan Medical University Hospital (CSMUH No: CS1-21128), conforming to the standards of the Declaration of Helsinki. Written informed consent was obtained from all participants prior to their inclusion in the study. The present study recruited a cohort of 50 young adults of Asian descent, aged between 20 and 30 years (mean age: 22.12 ± 2.2 years), comprising 27 females and 23 males. A subjective ocular examination, including assessments of stereopsis and visual acuity, was performed before refraction measurement. The distance visual acuities were measured at 6 m using the Snellen chart on the projector, and the results were converted into logMAR acuities for data analysis. The stereopsis of the participants was measured by the Titmus Stereo Fly test and recorded as seconds of arc. This study included participants with refractive errors ranging from −12.00 D to +0.50 D. The criteria for inclusion were based on established clinical standards for visual acuity (20/20 to 20/40) and stereopsis (60 arc seconds or better). These standards are widely used in clinical practice and ensured that participants had sufficient visual function for accurate refractive error measurement. Specifically, amblyopia was excluded, which is clinically defined as a visual acuity of 20/40 or worse and a difference in vision between eyes of two or more lines on a vision chart, with no apparent structural abnormality [25]. Furthermore, clinically normal stereoacuity should be less than 40 to 60 s of arc, depending on the test and the study cited [26]. Participants with a history of ocular pathology, strabismus, amblyopia, previous ocular surgeries, or use of orthokeratology lenses were excluded. The measurements were taken under standardized lighting conditions to minimize environmental variability.

### 2.2. Light Field Virtual Reality

**Figure 1:**
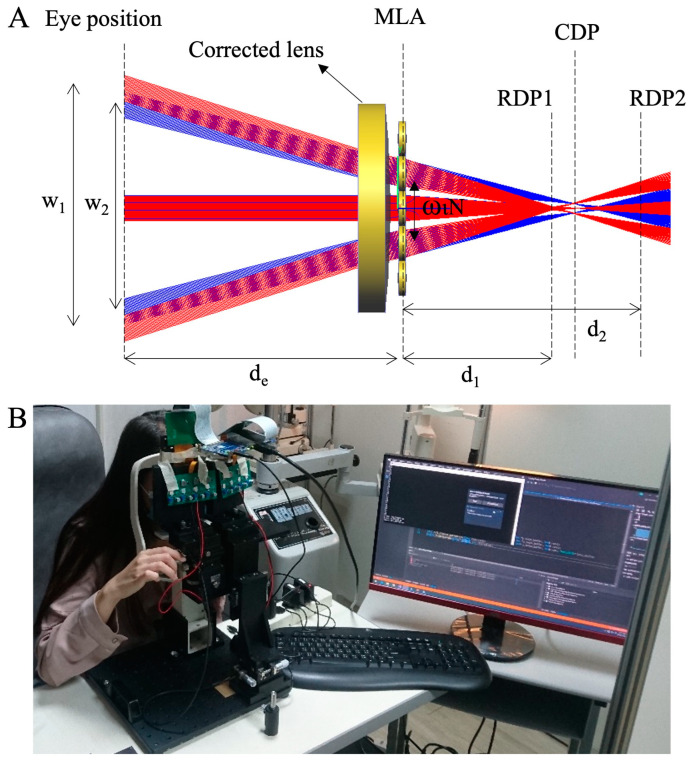
Schematic and photograph of the light field virtual reality (LFVR) system. (**A**) Schematic representation of the LFVR system’s eye box architecture. The system employed a microlens array (MLA) and a microdisplay to project a virtual reality image, with the central depth plane (CDP) and reconstructed depth plane (RDP) highlighted. (**B**) Photograph of the LFVR device.

Near-eye light field displays (NE-LFDs) is designed and constructed by Coretronic Corp. (Taiwan) using various plenoptic function-generating methods, including microlens arrays (MLAs). MLAs facilitate integral imaging (InIm), offering ultra-thin volumes, high optical efficiency, and simple hardware for full color production [27,28,29]. Figure 1 illustrates the principle underlying the proposed eye box mapping optimization method and a photograph of the device.

The reconstructed depth plane (RDP) is the plane where the image point resides, while the central depth plane (CDP) represents the native virtual image plane of the MLA [29,30]. The virtual images on RDP 1 and RDP 2 are inspected based on the reconstructed angle from each lenslet. Constructed depths can be extracted by considering the numbers of these lenslets (N) and the lens pitch (w_l_), within the boundaries of the eye box size ($w_{1}$ and $w_{2}$). To achieve vision correction, the image on the RDP2 plane should be transferred to the RDP1 plane, which falls within the myopia tolerance range. Figure 1 demonstrates that a corrected lens positioned near the MLA can redirect the rays from the eye to the MLA using reverse-ray tracing strategies, thereby mapping the eye box size without necessitating an alteration in MLA position. Concurrently, the power of the corrected lens facilitates depth shifting from $d_{2}$ to $d_{1}$ (Equation (2)). Consequently, the power (D) of the correction lens can map the modified eye box size under normal vision conditions, as delineated in Equations (1)–(3).

(1) $$\frac{w_{l}N}{w_{1}}=\frac{d_{1}}{d_{e}+d_{1}}\;\&\;\frac{w_{l}N}{w_{2}}=\frac{d_{2}}{d_{e}+d_{2}}$$

(2) $$D=\frac{1}{d_{2}}-\frac{1}{d_{1}}$$

(3) $$\frac{w_{2}}{w_{1}}=\frac{d_{1}(d_{e}+d_{2})}{d_{2}(d_{e}+d_{1})}$$

### 2.3. Testing Procedure

The study was structured over two days and the LFVR procedure was executed in the following manner:

Initially, the first focal line (F1) was examined. Throughout this phase, the left eye was occluded to facilitate the right eye’s examination. This process commenced following the removal of prescription glasses. The OFA-measured spherical prescription was then increased by +2.00 D as a baseline, followed by gradual decrement of the positive spherical prescription. The participant was asked to initiate looking at the clock diagram when the vision started to clear, and was queried about the clarity of lines in different directions. If a line appeared clearer in a specific direction, it was recorded as F1. Conversely, if all lines appeared equally clear, the interpretation was myopia without astigmatism. Subsequently, the second focal line (F2) was scrutinized. The positive spherical prescription was continuously reduced. Participants were prompted to focus on the line located 90 degrees from F1. As this line gained clarity, the refraction at this point was documented, providing the F2 refraction measurement.

Comparative analyses were conducted on the refractive measurements procured through each method. Sphere power (S), cylinder power (C), and axis (θ) measurements were transformed into spherical equivalent refraction (SE) and Jackson cross-cylinder (J_0_ and J_45_) values using the following formulas:

(1) SE = S + (C/2)
(2) J_0_ = −(C/2) × cos(2θ)
(3) J_45_ = −(C/2) × sin(2θ)

J_0_ symbolizes the powers at 90° and 180°. A negative J_0_ value identifies “against the rule” astigmatism, while a positive J_0_ value denotes “with the rule” astigmatism. J_45_ is indicative of oblique astigmatism, with a positive J_45_ value corresponding to a negative cylinder with an axis of 45° [31].

### 2.4. Statistical Analysis

In this study, data were analyzed using IBM SPSS Statistics version 25 (SPSS Inc., Chicago, IL, USA). Specifically, refractive data from the right eye were compiled and analyzed. The normality of the data was assessed using the Kolmogorov–Smirnov test. The results indicated that some datasets, including LFVR and J_0_, LFVR and J_45_, RET and J_0_, and RET and J_45_, did not follow normal distributions (*p* < 0.05). Consequently, we employed non-parametric methods, such as the Friedman test, to analyze these datasets. The Friedman test was used to compare the SE and two Jackson cross-cylinder values (J_0_ and J_45_) procured through disparate methods. Adjustments for pairwise comparisons were made using the Bonferroni correction [31].

To evaluate the concordance between LFVR and the other employed methods, both interclass correlation coefficients (ICC) and Bland–Altman analysis were utilized [32]. ICC values were interpreted as follows: 0–0.2 represented poor agreement, 0.3–0.4 indicated fair agreement, 0.5–0.6 denoted moderate agreement, 0.7–0.8 demonstrated strong agreement, and values exceeding 0.8 indicated near-perfect agreement [33,34]. Limits of agreement (LoA) were established as the mean difference ± 1.96 times the SD of the mean difference [35]. For all statistical analyses, a *p*-value less than 0.05 was considered statistically significant.

## 3. Results

A thorough analysis of the participants’ demographic characteristics was conducted. In terms of SE refraction error, as determined by autorefractor measurement, values spanned a broad range from +0.50 to −12.00 D (−4.46 ± 2.42 D) across the 100 examined eyes.

### 3.1. Evaluation of Refractive Components via LFVR and Other Clinical Methods

Table 1 presents the refractive components (SE, F1, F2, J_0_, and J_45_) obtained via LFVR, CFA, OFA, RET, and SR. The respective average (± SD) values of SE refraction, F1, and F2, as assessed by the five methods, were −6.33 ± 2.39 D, 5.96 ± 2.29 D, and −6.75 ± 2.69 D, respectively. The SE determined by LFVR was skewed toward more myopia by roughly 1.0 D to 1.5 D in comparison with the other four clinical instruments.

### 3.2. Correlation and Repeatability of Refractive Components Measured by LFVR versus Other Clinical Methods

Table 2 shows the ICCs for the refractive components (SE, F1, and F2) as measured by LFVR, CFA, OFA, RET, and SR. For SE, the calculated ICCs for comparisons between LFVR and CFA, OFA, RET, and SR were 0.852, 0.855, 0.827, and 0.847, respectively. For F1, the ICCs were 0.839, 0.838, 0.807, and 0.820, respectively. For F2, the corresponding values were 0.861, 0.867, 0.843, and 0.864, respectively. LFVR and the four clinical methods showed very good to excellent agreement in terms of the values of SE, F1, and F2 obtained (*p* < 0.01). The mean difference (bias) between the two visits, the repeatability of the focal lines of F1 and F2 as measured with LFVR, and the confidence intervals for the limits of agreement are reported in Table 3.

### 3.3. Agreement between LFVR and Other Clinical Methods

**Figure 2:**
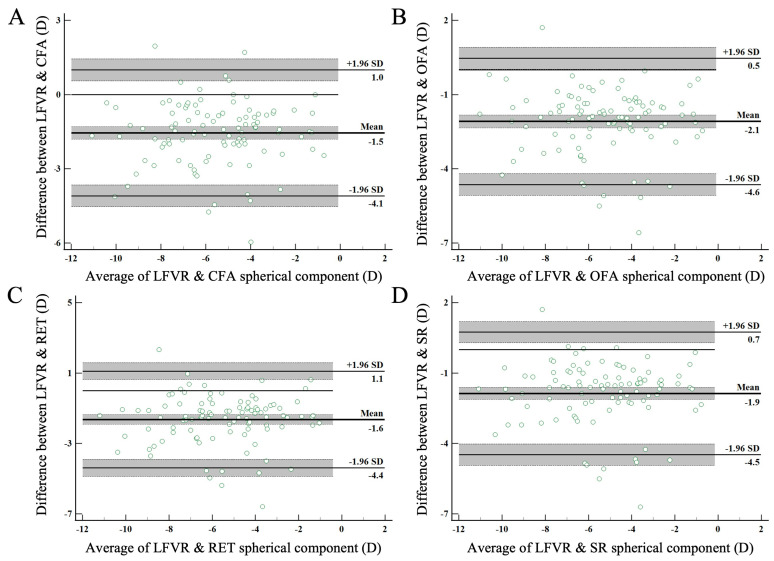
Bland–Altman plot comparing the mean spherical equivalent component across three instruments and the light field virtual reality system evaluated in this study. Each panel depicts the mean differences (MDs) and 95% limits of agreement (LoA), along with the confidence intervals for the LoA. The shaded areas represent the 95% confidence intervals. (**A**) Comparison between LFVR and CFA. The mean difference is −1.5 D, with 95% LoA from −4.1 D to 1.0 D. (**B**) Comparison between LFVR and OFA (open-field autorefractor). The mean difference is −2.1 D, with 95% LoA from −4.6 D to 0.5 D. (**C**) Comparison between LFVR and RET (retinoscopy). The mean difference is −1.6 D, with 95% LoA from −4.4 D to 1.1 D. (**D**) Comparison between LFVR and SR (subjective refraction). The mean difference is −1.9 D, with 95% LoA from −4.5 D to 0.7 D (CFA: closed-field autorefractor; D: diopter; LFVR: light field virtual reality; OFA: open-field autorefractor; RET: retinoscopy; SE: spherical equivalent; SR: subjective refraction).

The concordance of measurements between LFVR and other clinical instruments, as well as with established standards, was investigated using Bland–Altman analysis. Bland–Altman plots, depicted in Figure 2, illustrate the mean SE refraction, where the differences between methods are plotted against their mean values, thus providing the confidence intervals of the upper and lower limits of agreement (Figure 2A: LFVR and CFA; Figure 2B: LFVR and OFA; Figure 2C: LFVR and RET; Figure 2D: LFVR and SR) [36,37]. As displayed in Figure 2, the LFVR consistently indicated higher myopia (−1.80 D) in comparison to the other instruments. Specifically, the mean difference in SE between CFA and LFVR was −1.56 ± 1.30 D, corresponding to 95% limits of agreement of 2.54 D (Figure 2A). The mean difference in SE between OFA and LFVR was found to be −2.09 ± 1.30 D, with 95% limits of agreement at 2.55 D (Figure 2B). In relation to RET, the mean SE difference was −1.64 ± 1.40 D, resulting in 95% limits of agreement of 2.75 D (Figure 2C). Lastly, when comparing with SR, the mean SE difference was −1.87 ± 1.33 D, leading to 95% limits of agreement of 2.61 D (Figure 2D).

### 3.4. Correlation between Refractive Errors Measured by LFVR and Other Clinical Methods

**Figure 3:**
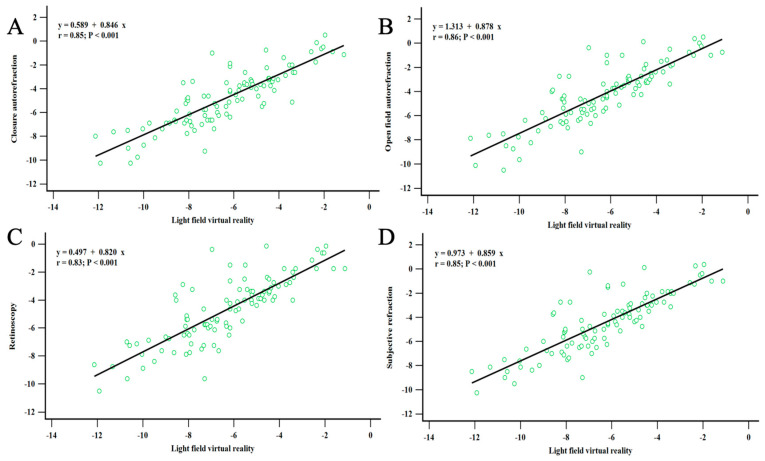
Comparative analysis of refraction error measured by the light field virtual reality system against four clinical instruments. (**A**) The correlation between LFVR and CFA, (**B**) the correlation between LFVR and OFA, (**C**) the correlation between LFVR and RET, and (**D**) the correlation between LFVR and SR. The LFVR exhibited a stronger correlation with the open-field autorefractor than the other three instruments, although correlations with all four measurements were substantial, highlighting LFVR’s reliability for refraction error assessment (CFA: closed-field autorefractor; D: diopter; LFVR: light field virtual reality; OFA: open-field autorefractor; RET: retinoscopy; SE: spherical equivalent; SR: subjective refraction).

Figure 3 illustrates a comparison between the refractive error measurements obtained by LFVR and other clinical instruments. A strong correlation was observed between the LFVR and CFA measurements (Pearson r = 0.85, *p* < 0.001) as depicted in Figure 3A. Similarly, LFVR and OFA measurements were strongly correlated (Pearson r = 0.86, *p* < 0.001), as indicated in Figure 3B. Furthermore, LFVR demonstrated a strong correlation with both RET and SR measurements (Pearson r = 0.83, *p* < 0.001; Pearson r = 0.85, *p* < 0.001, respectively), as shown in Figure 3C,D.

## 4. Discussion

The present study evaluated the LFVR system’s performance in assessing refractive error in comparison with the clinical gold standard methods, including RET, CFA, OFA, and SR. The outcomes indicated remarkable concordance among these methods concerning SE, F1, and F2 values. However, the LFVR system consistently showed an overestimation of myopia compared to RET and the other instruments. The Bland–Altman analysis revealed biases, with the least bias observed between LFVR and CFA. This overestimation can be attributed to instrument myopia, which causes an overactive accommodation during the detection phase, leading to more myopic measurements. Future modifications to the LFVR system could address this bias and improve its accuracy.

Previous work by Pujol et al. corroborated the acceptability of an electro-optical lens (EOL) system for refractive error correction, displaying considerable concurrence with traditional SR tests in the majority of adult participants [38,39]. Meanwhile, Goyal et al. utilized a tilted reflector to modulate the image presentation distance within a VR device, yielding an average discrepancy of −0.03 D [40]. Such findings attest to the significant agreement between the VR system and conventional SR examinations.

However, the analysis revealed that the SEs and the F1 and F2 measurements acquired via the LFVR system were more myopic compared to the other four assessments, with a range from −1.50 D to −2.00 D. This deviation may be attributable to instrument myopia, a phenomenon in which an overactive accommodation during the detection phase leads to the perception of approaching objects [15,41]. The literature has underscored that optometric instruments can elicit myopic shifts, with errors spanning from 0.50 D to 5.00 D [16,42]. The LFVR system, given its resemblance to a closed machine, could prompt an overcorrection and introduce a myopic bias. Adjusting for the refraction errors elicited by instrument accommodation brings the measurements more in line with conventional refraction assessments. The alignment between LFVR and objective refraction measurements parallels prior research [40]. Future modifications to the instrument can potentially compensate for the excess refractive errors measured, allowing the measured refraction to more accurately represent the actual condition.

Accommodation can be influenced by a range of factors, including refractive error magnitude, monocular or binocular vision, age, environmental brightness, and psychological factors [43,44,45,46]. This study focused on adolescent subjects with low to moderate myopic refractive errors. This demographic is known to demonstrate higher accommodative responses [47] and more significant accommodative lag compared to emmetropic or hyperopic groups [48]. The interplay between accommodation and phoria status also suggests an enhanced vergence accommodation in individuals with higher phoria [49]. Further, both the examinee’s and the examiner’s familiarity with the process can impact instrument-induced myopia [46].

Prolonged use of VR systems can precipitate fatigue, thereby affecting visual function. Notably, reduced accommodation amplitude, exophoric deviation, and compromised stereoscopic function have been observed following prolonged use of head-mounted VR [50,51]. In this study, the participants were novice refractive examiners using the LFVR system. The movement of visual targets potentially influenced the accommodative response, resulting in extended examination durations, ocular fatigue, and induced myopic shifts [52,53]. This, combined with the unfamiliarity and anxiety experienced by the participants, likely affected the accuracy of the results. The net result was elongated examination times, ocular fatigue, and instrument-induced myopic shifts, thereby potentially compromising the accuracy of the findings. Future studies should include a training session for participants to familiarize themselves with the LFVR system before the actual measurements. To mitigate the effects of fatigue, it is recommended to include regular breaks and shorten the duration of each measurement session.

Visual performance is significantly influenced by factors such as resolution, color saturation, contrast, and brightness [54,55]. Higher spatial frequencies may result in blurred images due to impaired accommodation [56], and objects with a gray hue are often more perceptible than brightly colored ones in ambient lighting conditions [57]. Contrast sensitivity, closely tied to color recognition, can be augmented by improved resolution, thereby enhancing clarity and providing more precise measurements [58,59]. In this study, the color saturation and contrast of the examined image were not fully equivalent to the realistic clock dial chart, potentially introducing a margin of error in these results [60]. Enhanced resolution could increase contrast sensitivity and refine the subject’s view of the target, facilitating more accurate outcomes.

Accurate refractive examinations are heavily reliant on depth perception, a complex interplay of binocular vision, accommodation, and stereopsis [61]. The integration of these elements impacts distance judgments. Studies have demonstrated that both stereopsis and luminance of stimuli influence depth judgment, with brighter visual targets resulting in distance underestimations and darker targets leading to overestimations [60,62]. To minimize potential bias in the present study, stereopsis was established as a selection criterion, and consistent illumination levels were maintained for all participants. However, it should be underscored that the subjects were in an uncorrected refractive state during measurement. Prior studies suggest that correction status of refractive errors can influence stereopsis, thereby affecting depth perception and distance judgments [63]. To address the issues identified, the present study proposes increasing the sample size and refractive range in future studies to validate the LFVR system’s performance across diverse populations. Adjusting internal system parameters and implementing adaptive algorithms can help to reduce the vergence-accommodation conflict and enhance measurement accuracy. Developing advanced optical designs, such as light field displays or varifocal systems, will also help to mitigate this conflict. Additionally, providing training sessions for participants and incorporating regular breaks during measurements can reduce anxiety and fatigue, further improving the accuracy and reliability of the LFVR system in clinical practice and research.

The LFVR system employed in this study harnessed light field technology, adjusting the microlenses in the device to manipulate the image size and craft a VR scenario within a defined space. It should be noted that VR devices with low screen resolution can induce a screen door effect, thereby rendering pixels noticeable and leading to blurred images [64,65]. Interestingly, these findings suggest that the arrangement of microlenses can influence the sharpness of the image perceived by the user, particularly among individuals with higher refractive errors. Despite all participants having normal visual acuity, the data could potentially have been impacted by the device used in testing.

Vergence and accommodation are two important ocular functions that interact to preserve clear vision. Under natural conditions, these functions tend to be isometric and coincide with the demand line, known as Donder’s line [66]. However, in VR systems or 3D stereoscopic displays, the focal adjustment of the eyes remains fixed on the screen, while vergence fluctuates according to the depth of the stereoscopic image, thereby creating conflict and misalignment between the two functions. Such discrepancies can trigger symptoms like dizziness and nausea and may introduce errors when assessing refractive errors [52,67]. Notably, usage of the head-mounted VR system resulted in a significant rise in fatigue and discomfort scores. A conspicuous shift was observed in myopia, which might be attributable to the discord between accommodation and vergence [53,67]. As the conflict primarily occurs during binocular viewing, where the eyes must use both accommodation and vergence to focus on objects at different depths, the present study focused on monocular viewing conditions. In monocular viewing, the eye only needs to use accommodation to focus on the target, which significantly reduces the impact of vergence conflicts. Additionally, the LFVR system employed in this study integrated advanced light field technology, which adjusted the microlens array to provide sufficient depth cues. This alignment ensured that the accommodation and vergence functions were maintained on the same plane, thereby significantly reducing the discomfort associated with vergence-accommodation conflicts. Consequently, the potential influence of this effect on the study results is likely minimal.

The present study has several limitations that should be noted. Firstly, the sample was limited to individuals with myopia and mild-to-moderate astigmatism, which restricted the ability to assess hyperopia and high astigmatism. Future studies should include a larger, more diverse sample to validate the LFVR system’s performance across different demographics. Additionally, the clock dial chart used differed in resolution from those in clinical practice, complicating the detection of low astigmatism. Expanding the measurement range beyond ±14.00 D and including binocular measurements would provide a more comprehensive evaluation. The present study did not account for the potential impact of prolonged VR system use on visual comfort and measurement accuracy, highlighting the need for protocols to mitigate visual fatigue in future research.

Moreover, the reliance on light field technology and microlens arrays introduces calibration complexities. Standardized calibration protocols and robust maintenance procedures are needed to ensure reliability. The exclusion of participants with ocular pathologies may have introduced selection bias, limiting the findings’ applicability to those with such conditions. Although instrument-induced myopia was observed, further exploration on adjusting or mitigating this effect is required. Future studies should implement calibration processes, adaptive algorithms, and advanced optical designs to address this issue. Training sessions and regular breaks during measurements can also improve accuracy. Addressing these limitations will be crucial for establishing the LFVR system’s robustness and applicability in diverse clinical settings.

## 5. Conclusions

In conclusion, this study demonstrates that the LFVR system has substantial agreement and high reproducibility for measuring refractive error, as evidenced by strong ICCs with clinical gold standard methods. However, the system showed a consistent overestimation of myopia, which was least pronounced when compared to CFA. These findings underscore the LFVR system’s potential as a reliable tool for refractive error assessment in optical care, with the caveat of addressing the current limitations related to myopia overestimation.

## Figures and Tables

**Table 1:** Comparison of the refractive components between light field virtual reality, closed-field autorefractor, open-field autorefractor, retinoscopy, and subjective refraction.

	SE (D)	J_0_ (D)	J_45_ (D)	F1 (D)	F2 (D)
LFVR	−6.33 ± 2.39	0.27 ± 0.59	−0.02 ± 0.32	−5.96 ± 2.29	−6.75 ± 2.69
CFA	−4.77 ± 2.37	0.36 ± 0.50	−0.03 ± 0.31	−4.20 ± 2.26	−5.34 ± 2.54
OFA	−4.25 ± 2.45	0.35 ± 0.47	−0.04 ± 0.30	−3.70 ± 2.33	−4.80 ± 2.61
RET	−4.70 ± 2.37	0.45 ± 0.57	0.00 ± 0.23	−4.10 ± 2.21	−5.29 ± 2.60
SR	−4.46 ± 2.42	0.43 ± 0.54	−0.04 ± 0.29	−3.87 ± 2.22	−5.06 ± 2.68

LFVR: light field virtual reality; CFA: closed-field autorefractor; D: diopter; F1: back focal line; F2: front focal line; OFA: open-field autorefractor; RET: retinoscopy; SE: spherical equivalent; SR: subjective refraction.

**Table 2:** Comparison of the interclass correlation coefficients, 95% limits of agreement, and correlation of the refractive components of different methods against the light field virtual reality.

		ICC	95% CI	*p*-Value
LFVR-CFA	SE	0.852	0.79 to 0.90	<0.01
	F1	0.839	0.77 to 0.89	<0.01
	F2	0.861	0.80 to 0.90	<0.01
LFVR-OFA	SE	0.855	0.79 to 0.90	<0.01
	F1	0.838	0.77 to 0.89	<0.01
	F2	0.867	0.81 to 0.91	<0.01
LFVR-RET	SE	0.827	0.75 to 0.88	<0.01
	F1	0.807	0.73 to 0.87	<0.01
	F2	0.843	0.77 to 0.89	<0.01
LFVR-SR	SE	0.847	0.78 to 0.89	<0.01
	F1	0.820	0.74 to 0.88	<0.01
	F2	0.864	0.80 to 0.91	<0.01

CFA: closed-field autorefractor; CI: confidence interval; F1: back focal line; F2: front focal line; LFVR: light field virtual reality; OFA: open-field autorefractor; RET: retinoscopy; SE: spherical equivalent; SR: subjective refraction.

**Table 3:** Test of repeatability of the light field virtual reality in 30 eyes of 15 individuals.

	First Focal Line		Second Focal Line	
	ICC (%)	95% CI	ICC (%)	95% CI
LFVR	88.8	0.85 to 0.92	97.5	0.97 to 0.98

CI: confidence interval; LFVR: light field virtual reality.

## Data Availability

The data that support the findings of this study are available upon request from the corresponding author. The data are not publicly available due to privacy or ethical restrictions.
